# Transurethral thulium laser resection via ureterorenoscope for pediatric low-grade urothelial carcinoma of the bladder: a case report

**DOI:** 10.1186/s12893-026-03534-7

**Published:** 2026-02-02

**Authors:** Pinyao Liang, Jianheng Chen, Jian Shen, Yumin Wang, Junxiong Li, Jingbo Qin, Peng Gu, Xiaodong Liu

**Affiliations:** 1 Second Department of Urology, The First Affiliated Hospital of Kunming Medical University, Kunming, China; 2 Department of Pathology, The First Affiliated Hospital of Kunming Medical University, Kunming, China

**Keywords:** Bladder urothelial carcinoma, Children, Thulium laser, Ureteroscopy, Case report

## Abstract

**Objective:**

To report a case of pediatric bladder urothelial carcinoma treated with transurethral thulium laser resection and, based on a literature review, to discuss its clinical characteristics, diagnostic considerations, and surgical strategies adapted to the unique anatomy of the pediatric urethra.

**Methods:**

The clinical data of a child with bladder urothelial carcinoma admitted to the Second Department of Urology, The First Affiliated Hospital of Kunming Medical University in October 2024 were retrospectively analyzed.

**Result:**

A 9-year-old boy presented with transient, painless gross hematuria as the primary clinical manifestation. Bladder tumor was diagnosed via imaging. He subsequently underwent transurethral thulium laser resection of the bladder tumor via ureteroscopy under general anesthesia. The procedure was successful, and immediate postoperative intravesical chemotherapy was administered. Histopathological examination confirmed low-grade papillary urothelial carcinoma of the bladder. The patient has since received regular postoperative intravesical chemotherapy. Follow-up at one year showed no evidence of tumor recurrence on ultrasound.

**Conclusion:**

Bladder urothelial carcinoma is exceedingly rare in children. Raising awareness and vigilance among healthcare providers and families is essential for early diagnosis. In cases where standard adult instruments are unsuitable, transurethral thulium laser resection via a ureterorenoscope represents a safe and feasible adaptive technique for pediatric low-grade non-muscle-invasive urothelial carcinoma. This report offers a practical reference for the management of such rare cases.

## Introduction

Bladder urothelial carcinoma is a common urological malignancy but occurs infrequently in children. Due to its very low incidence in the pediatric population, standardized treatment protocols and follow-up strategies are currently lacking. The Second Department of Urology, The First Affiliated Hospital of Kunming Medical University admitted a child with bladder urothelial carcinoma in October 2024. The patient successfully underwent transurethral thulium laser resection of the bladder tumor via ureteroscopy under general anesthesia.

## Clinical data

### General information

A 9-year-old boy was admitted on October 12, 2024, due to “transient gross hematuria for 10 days.” Ten days prior to admission, the child experienced an episode of transient gross hematuria without apparent cause. There were no associated symptoms such as urinary frequency, urgency, dysuria, chills, fever, or low back pain. He sought medical attention at an outside hospital where investigations were performed, and symptomatic supportive treatment was administered. Then, he was referred to our hospital for further diagnosis and treatment. Furthermore, his past medical history was unremarkable, and physical examination revealed no significant abnormalities.

### Laboratory and imaging findings

**Fig. 1:**
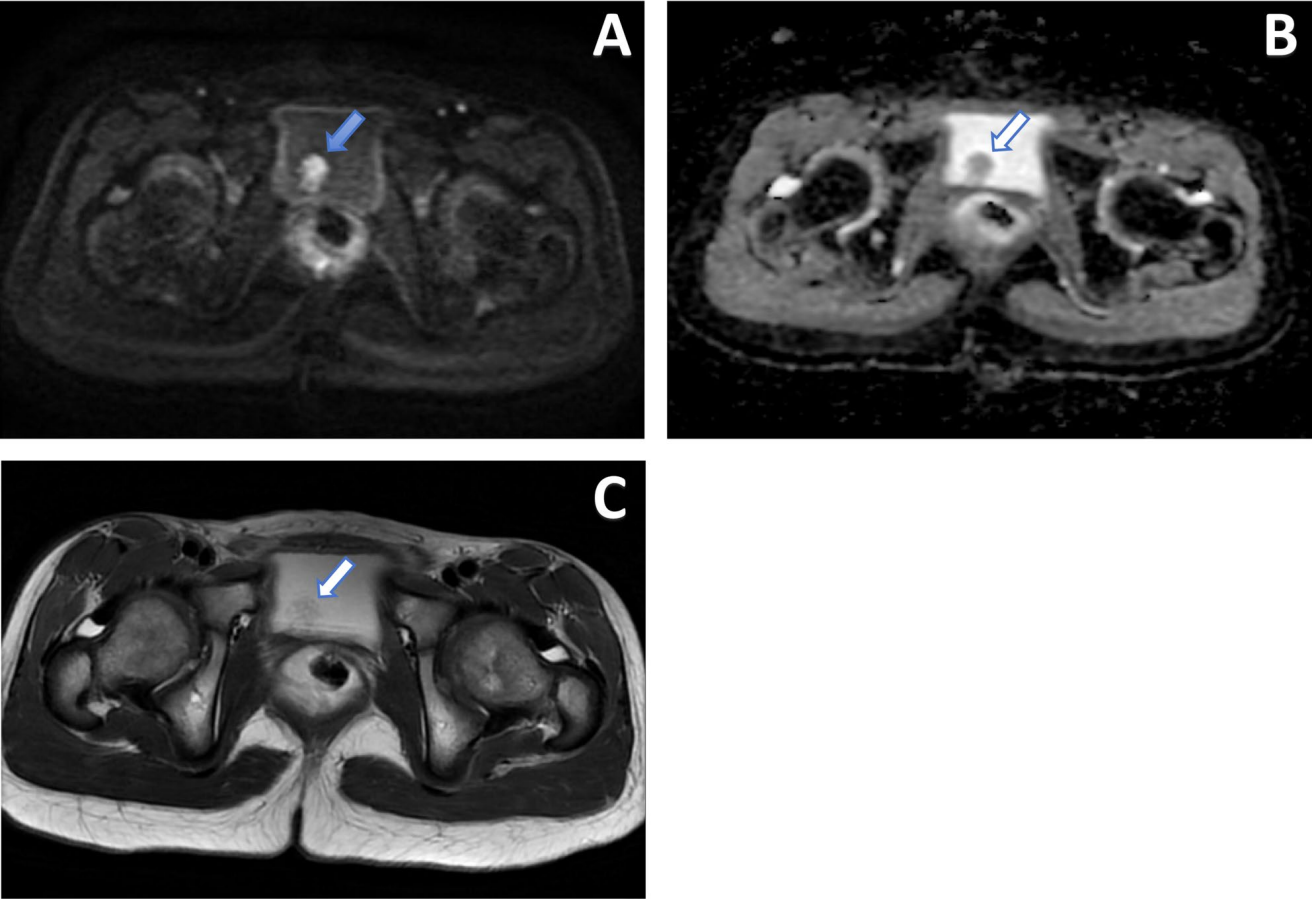
MRI findings of a bladder mass on the right lateral wall. **A** Axial diffusion-weighted image (DWI) shows high signal intensity. **B** Axial apparent diffusion coefficient (ADC) map shows corresponding low signal intensity. **C** Axial T2-weighted image (T2WI) shows an intermediate signal intensity mass (arrows)

Routine blood tests, coagulation function, and biochemical profiles showed no significant abnormalities. Urinalysis revealed red blood cells (++). Ultrasound demonstrated a hyperechoic mass on the right lateral wall of the bladder, measuring approximately 2.1 cm × 1.8 cm × 1.3 cm, with regular morphology and mobility on positional change. MRI revealed an irregular nodule on the right bladder wall, displaying slightly short T2 and isointense T1 signals, high signal on DWI, low signal on ADC, and post-contrast enhancement. The lesion measured about 1.3 cm × 1.4 cm × 1.0 cm, with locally indistinct borders from the bladder wall (Fig. 1). These imaging features are suggestive of a bladder-occupying lesion, likely neoplastic in nature.

### Surgical procedure

On the second day after admission, the patient underwent transurethral thulium laser resection of bladder tumor via a ureterorenoscope under general anesthesia. Positioned in lithotomy, the perineum was routinely sterilized and draped. A ureteroscope (F7.5) was inserted transurethrally into the bladder under direct vision. Intraoperatively, a single, pedunculated, cauliflower-like tumor measuring approximately 13 × 14 mm was visualized on the right lateral wall near the bladder neck (at the 10 o‘clock position). No other lesions were noted in the remainder of the bladder mucosa. The tumor was completely resected using a 200 μm thulium laser fiber at 25 W power, with a 5 mm resection margin from the tumor base. Due to the tumor’s challenging location and restricted operating space, vaporization extended only to the superficial detrusor muscular layer, and an adequate deep muscle sample could not be obtained for pathological staging. All resected specimens were retrieved using grasping forceps. After confirming hemostasis, the scope was withdrawn. A 12Fr double-lumen urinary catheter was indwelled, followed by immediate postoperative intravesical instillation of pirarubicin. The patient was transferred to the recovery room in stable condition.

## Results

**Fig. 2:**
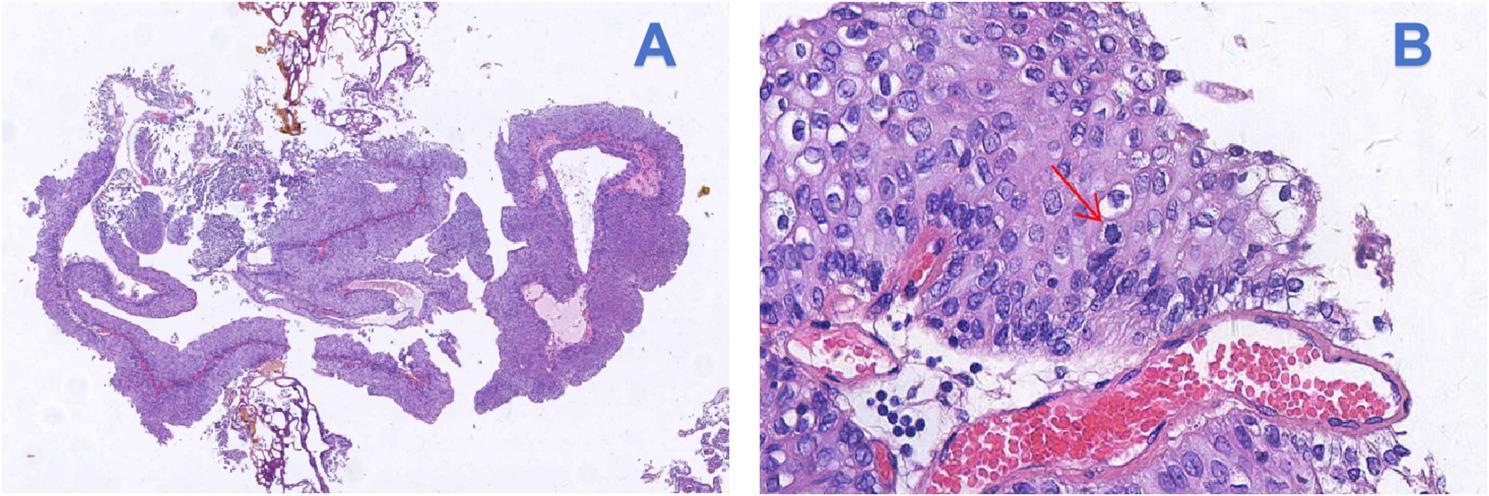
Pathological diagnosis of low-grade papillary urothelial carcinoma (H&E). **A** Low-power view (×40) reveals papillary architecture with fibrovascular cores. **B** High-power view (×200) shows tumor cells with occasional pathological mitotic figures (arrows)

**Fig. 3:**
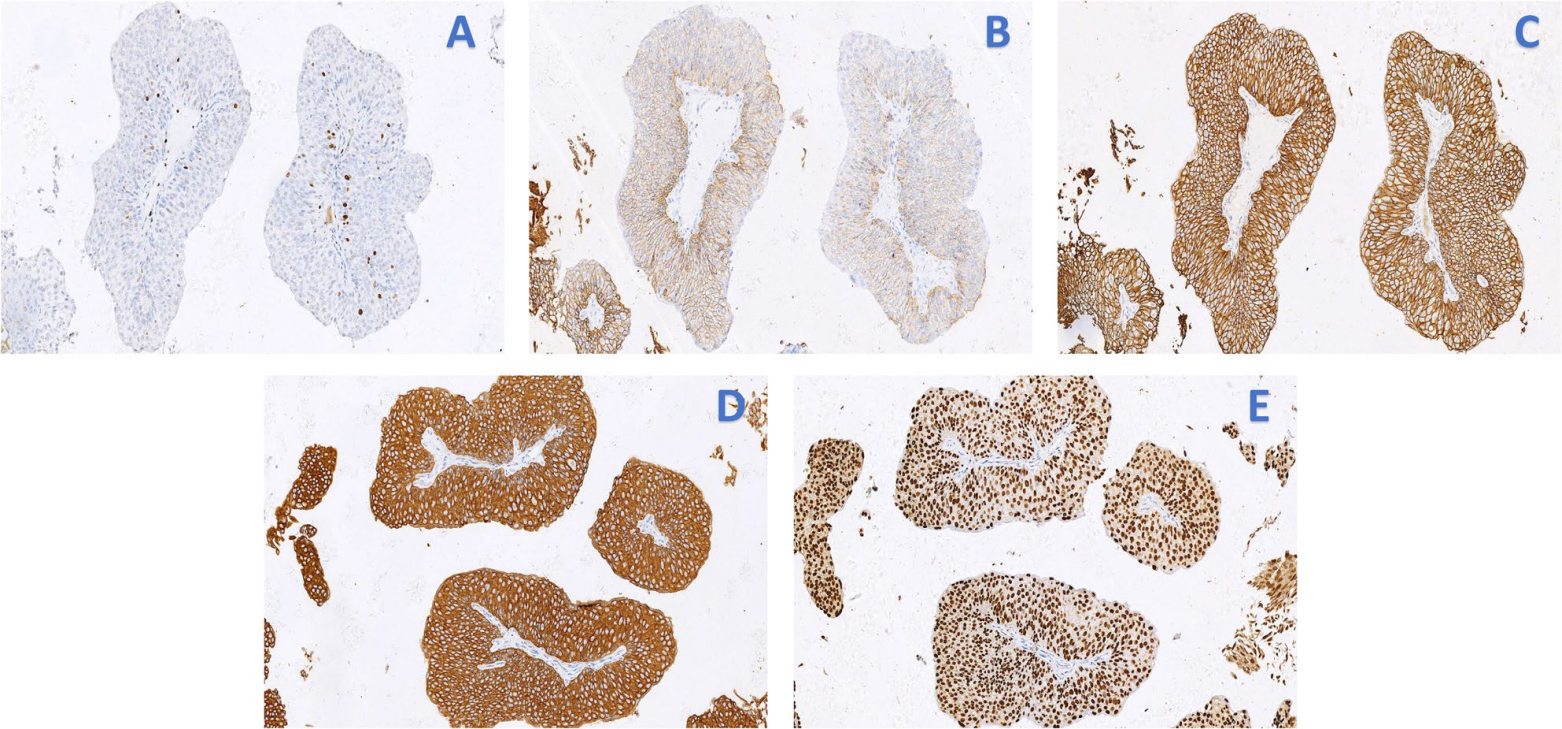
Immunohistochemical profiling of low-grade papillary urothelial carcinoma. The tumor cells demonstrate: **A** a Ki-67 proliferation index of approximately 5%. **B** focal weak positivity for CD44. **C** positive membrane staining for E-cadherin. **D** diffuse positivity for CK7. **E** strong nuclear positivity for GATA3. (All immunohistochemical stains were developed with diaminobenzidine chromogen and counterstained with hematoxylin; original magnification ×200)

The procedure was completed successfully without intraoperative complications. The patient’s postoperative recovery was uneventful, and the bladder irrigation fluid remained clear. The urethral catheter was removed on postoperative day 1, and the child was discharged. Postoperative hematoxylin and eosin (H&E) staining revealed a urothelial proliferative lesion with focal papillary structures (Fig. 2). Immunohistochemistry (IHC) results were as follows: CK (diffuse strong +), VIM(-), CK7(+), CK20(-), Ki67(+) approximately 5%, UPK2(-), CD44 (focal weak +), GATA3(+), Her-2(0), E-cad(+), P53(-), P63(+) (Fig. 3). The Pathological diagnosis was low-grade papillary urothelial carcinoma of the bladder. Postoperative intravesical therapy with Pirarubicin (20 mg diluted in 30 ml of 5% glucose solution) was administered monthly for a total of 12 cycles. As the patient and family declined cystoscopic surveillance, follow-up for this case primarily relied on urinalysis and ultrasound examinations, currently performed every 3 months. The patient has been followed for one year postoperatively, with no signs of tumor recurrence.

## Discussion

Bladder cancer, as the most common malignant tumor in urology, predominantly affects middle-aged and elderly individuals, while being relatively rare in young patients. Bladder urothelial carcinoma is reported to occur more frequently in males. Its incidence in individuals under 40 years old ranges from 1% to 2.4%, whereas among adolescents and children under 20 years old, the incidence is notably lower at 0.1%-0.4% [1]. Pediatric cases are primarily characterized by non-invasive, low-grade tumors exhibiting slow progression and favorable prognosis [2]. With advancing age, the incidence of high-grade urothelial carcinoma gradually increases, accompanied by greater invasive potential [1].The development of bladder cancer in adults is associated with risk factors such as smoking, occupational exposures, chemotherapeutic agent administration, and chronic infections. In contrast, the relationship between smoking and pediatric bladder carcinoma remains to be further elucidated, while other adult risk factors are generally not considered applicable due to age-related constraints [3].

Bladder tumors typically present with painless gross hematuria or bladder irritation symptoms as the initial manifestation. Some patients may also experience low back pain and dysuria, while a minority present solely with microscopic hematuria. Isolated, painless gross hematuria is the most common symptom of bladder tumors in children [4, 5]. However, due to a generally lower level of suspicion for bladder malignancy in this age group, it is often overlooked. When a child presents with gross hematuria for the first time, after excluding conditions such as urinary tract infection, urolithiasis, and nephritis, a high suspicion for a urological malignancy should be raised. Prompt investigation is crucial for early diagnosis. In the case presented here, the child sought immediate medical attention after an episode of transient gross hematuria and was diagnosed with a bladder tumor following comprehensive imaging studies. We contend that due to the very low incidence of pediatric bladder cancer, healthcare professionals often lack sufficient vigilance and awareness, leading to potential misdiagnosis or missed diagnosis. The occurrence of malignant tumors is increasingly observed in younger populations. According to World Health Organisation, 2025 statistics, approximately 400,000 children and adolescents aged 0–19 are diagnosed with cancer globally each year [6]. Therefore, enhancing the early diagnosis rate of pediatric bladder cancer hinges on improving the recognition and vigilance of both healthcare providers and family members regarding this disease. Strengthening education for families and society is paramount.

Cystoscopy with biopsy is the most reliable invasive method for diagnosing bladder tumors. However, due to its requirement for general anesthesia and the risk of potential injury to the immature urethra in children, its clinical use in this population is relatively limited. Ultrasound, being non-invasive, can detect 85%-100% of bladder tumors [7]. Owing to its convenience and high diagnostic yield, it should be prioritized as the initial screening modality for pediatric bladder tumors. Urine cytology is also non-invasive, but its sensitivity for well-differentiated tumors is only 6%-38% [3]. Since pediatric bladder cancers are predominantly low-grade tumors, it is not recommended for routine diagnosis or postoperative surveillance in children with bladder cancer [8]. In this case, urine cytology was not performed. Ultrasound revealed a slightly hyperechoic mass in the bladder. Subsequent MRI demonstrated an irregular nodule on the right lateral wall, showing slightly short T2 and isointense T1 signals, high signal on DWI, and low signal on ADC, which are imaging features consistent with bladder cancer. The child then underwent immediate transurethral thulium laser resection of the bladder tumor via ureteroscopy. This approach effectively achieved both diagnosis and treatment promptly, minimizing the discomfort associated with repeated biopsy procedures and reducing the risk of urethral injury in the child.

Treatment options for bladder cancer include transurethral resection of bladder tumor (TURBT), radical cystectomy, partial cystectomy, and chemotherapy. Both adult and pediatric bladder cancer management requires selecting different surgical approaches based on the tumor’s pathological grade and stage. However, the majority of pediatric bladder cancers are papillary urothelial neoplasms of low malignant potential (PUNLMP) or low-grade urothelial carcinoma. Rezaee et al. [4] reported in a systematic review that approximately 93.4% of affected children have low-grade tumors. In alignment with this finding, Saltsman et al. [5] confirmed in a large case series that about 91% of young patients present with non-invasive disease. Given this biological profile, TURBT serves as the primary therapeutic approach. It has been reported as the initial treatment in over 95% of published cases [4], a finding consistent with the result from Saltsman et al. [5] that 91% of tumors were amenable to TURBT. Additionally, pediatric resectoscopes are available; these low-Fr instruments are suitable for use in children.

The thulium laser has a wavelength of approximately 2 μm, which is very close to the absorption peak of water (1.94 μm), allowing its energy to be efficiently absorbed by both water and tissue. This physical property endows it with excellent vaporization and cutting capabilities, enabling continuous and precise energy delivery, resulting in a smooth resection plane, reliable hemostasis, and the formation of a thin coagulated eschar. Moreover, this coagulated layer maintains a relatively low temperature, causing minimal thermal damage to bladder tissue. This characteristic is particularly beneficial for reducing the risk of intramural ureteral stricture following resection of tumors located near the ureteral orifice [9, 10]. Collectively, these features ensure clear surgical visibility and controlled cutting depth, which not only helps reduce tumor seeding but also improves the quality of pathological specimens. This supports accurate tumor grading and staging and ultimately lowers the risk of repeat resection due to incomplete removal or staging uncertainty [10–13]. Compared to plasma kinetic resection, thulium laser surgery demonstrates advantages in terms of postoperative catheter indwelling time and hospital stay. Peng Xiaowen et al. [14] reported that, compared with transurethral plasma kinetic resection for non-muscle-invasive bladder tumors, transurethral thulium laser surgery showed superior outcomes in intraoperative bleeding control, with lower rates of bladder perforation and obturator nerve reflex, indicating better overall surgical efficacy. Zhong Xin et al. [15] further confirmed that, compared with conventional transurethral resection of bladder tumor (TURBT), thulium laser resection significantly reduces intraoperative blood loss, irrigation time, catheter indwelling time, complication rates, and levels of inflammatory factors. Furthermore, as the 2 μm thulium laser generates no electrical current locally during resection, it does not stimulate the obturator nerve and can effectively prevent obturator nerve reflex, thereby further reducing intraoperative and postoperative related complications [13, 16–18]. Since dedicated pediatric resectoscopes are seldom available, and conventional adult resectoscopes are generally too large to safely traverse the narrower pediatric urethra, transurethral thulium laser resection via ureteroscopy was selected for this patient. The surgery proceeded smoothly and was completed within 40 min. Nevertheless, this approach presents certain technical limitations: the ureteroscope does not consistently ensure adequate irrigation flow, maintain stable bladder distention, or provide optimal visual clarity throughout the procedure. To address these issues, some authors have proposed the use of a peel-away sheath as a working channel for the ureteroscope during tumor resection, which may improve fluid management and visual field stability [19]. However, given the rarity of pediatric bladder cancer, the feasibility and safety of the peel-away sheath in this population remain to be further evaluated in clinical practice.

The pathological grade, stage, and prognosis of pediatric bladder cancer are generally favorable. Most cases are PUNLMP or low-grade urothelial carcinoma, with high-grade urothelial carcinoma being less common. Tumor invasion depth typically does not extend beyond the lamina propria; invasion into the muscularis propria is exceedingly rare [8]. Therefore, postoperative adjuvant intravesical therapy (chemotherapy or immunotherapy) is usually not required. However, children with high-grade urothelial carcinoma remain at risk for recurrence and progression and may benefit from intravesical therapy [3]. Regular postoperative follow-up with urinalysis and ultrasound is essential. Since cystoscopy usually necessitates general anesthesia and large case series have shown a relatively low recurrence rate (approximately 8%) for non-invasive carcinoma in children and young adults [5], surveillance cystoscopy is generally reserved for cases where clinical symptoms or ultrasound findings raise suspicion of recurrence [7, 20]. Although the patient was diagnosed with low-grade papillary urothelial carcinoma, the tumor location posed spatial constraints during surgery, limiting the resection depth to the superficial muscular layer. Furthermore, no definitive muscularis propria was identified in the submitted specimens, preventing accurate assessment of muscle invasion. Additionally, studies have indicated that a certain proportion (approximately 17%) of bladder tumor specimens from young patients may be downgraded upon expert pathological review [5]. Considering that pediatric low-grade urothelial carcinoma, while predominantly non-muscle-invasive, presented in this case with limitations in intraoperative assessment and potential diagnostic uncertainty, intravesical chemotherapy was administered after obtaining informed consent from the child’s guardians to mitigate the risk of recurrence. The specific regimen consisted of pirarubicin 20 mg dissolved in 30 mL of 5% glucose solution, instilled into the bladder via a urethral catheter once monthly for a total of 12 cycles. To date, the patient has completed the entire course of instillations, and one-year postoperative ultrasound follow-up has shown no evidence of tumor recurrence. It must be emphasized that this intravesical chemotherapy regimen represents an individualized decision based on the specific circumstances of this case. For pediatric low-grade urothelial carcinoma, the necessity of adjuvant intravesical therapy, along with the optimal drug selection, treatment duration, and instillation frequency, lacks robust support from high-level evidence. While the monthly instillation schedule adopted in this protocol aimed to reduce urethral manipulation, its long-term efficacy in preventing recurrence compared to conventional regimens remains unestablished. Furthermore, intravesical chemotherapy itself carries inherent limitations, including potential local adverse effects such as chemical cystitis and lower urinary tract irritation symptoms. Consequently, the therapeutic approach described here should be regarded merely as a reference for clinical decision-making. Its long-term efficacy and safety profile necessitate further validation through extended follow-up and observation in larger patient cohorts.

## Conclusion

In summary, pediatric bladder cancer is exceedingly rare. Enhancing awareness and vigilance among healthcare professionals, families, and society regarding this disease is paramount for achieving early diagnosis and timely treatment. Currently, there is currently no standardized protocol for the treatment and follow-up of pediatric bladder cancer. Given the unique anatomical considerations of the pediatric urethra, conventional adult surgical instruments are often not suitable. The transurethral thulium laser resection via ureteroscopy employed in this report represents a safe and feasible adaptive technique for pediatric bladder urothelial carcinoma. Further clinical practice and research are required to explore and optimize diagnostic and therapeutic strategies for the pediatric population.

## Data Availability

The data supporting the findings of this case report are available within the article.

## Abbreviations

TURBT: Transurethral resection of the bladder tumor
IHC: Immunohistochemistry
PUNLMP: Papillary urothelial neoplasms of low malignant potential
